# Ultrasonic Influence on Macrostructure and Mechanical Properties of Friction Stir Welded Joints of Al/Mg Sheets with 2 mm Thickness

**DOI:** 10.3390/ma17164044

**Published:** 2024-08-14

**Authors:** Jialin Yin, Jie Liu, Chuansong Wu

**Affiliations:** MOE Key Laboratory for Liquid-Solid Structure Evolution and Materials Processing, Institute of Materials Joining, Shandong University, Jinan 250061, China; yinjialin@mail.sdu.edu.cn (J.Y.); 202134172@mail.sdu.edu.cn (J.L.)

**Keywords:** friction stir welding, ultrasonic vibration, thin sheet, Al alloy, Mg alloy, macrostructure, mechanical property

## Abstract

Friction stir welding (FSW) and ultrasonic vibration enhanced FSW (UVeFSW) experiments were conducted by using 6061-T6 Al alloy and AZ31B-H24 Mg alloy sheets of thickness 2 mm. The suitable process parameters windows were obtained for the butt joining of Al/Mg sheets. The effect of ultrasonic vibration on the macrostructure and mechanical properties of the dissimilar joints was studied. The results showed that the width of the weld nugget zone (WNZ) was enlarged to some extent and the hardness distribution in WNZ was more uniform in UVeFSW. In addition, the application of ultrasonic vibration effectively promoted the interpenetration degree of dissimilar materials in the WNZ so that the mechanical interlocking on the bonding interface of dissimilar Al/Mg materials was enhanced. The facture positions were changed from the bonding interface in FSW to the boundary between WNZ and the thermo-mechanical affected zone, and the ductile fracture zone was expanded. The highest ultimate tensile strength was 205 MPa at the process parameters set of 1200 rpm–50 mm/min in UVeFSW in this experiment. The average ultimate tensile strength of FSW/UVeFSW joints was 172.3 MPa and 184.4 MPa, respectively, and the average ultimate tensile strength was increased by 7.02% with the introduction of ultrasonic vibration.

## 1. Introduction

With the increasing urgency to achieve the goal of emission reduction and carbon neutrality, structural lightweight has become an important issue in manufacturing transportation vehicles at present. As lightweight alloys with excellent properties, Al alloys and Mg alloys have become important materials for manufacturing various components of structures. Recently, in order to fully realize the advantages of multi-material structures, the demand for hybrid structures of Al/Mg alloys has gradually increased [1,2]. However, due to the differences in crystal structure and physical–chemical properties of Al and Mg alloys, it is difficult to use fusion welding processes for achieving the high-quality and high-performance joints of Al-to-Mg. Friction stir welding (FSW), as a solid-state welding process, has obvious advantages in making dissimilar Al-to-Mg joints with excellent performance [3,4].

Brittle and hard intermetallic compound phase in Al-Mg dissimilar joint is one of the important factors leading to joint cracking [5,6]. Controlling the formation and distribution of intermetallic compounds has become a significant way to improve the properties of joints [1,7].

In order to further improve the strength and performance of FSWelded Al/Mg joints, the FSW process has been modified by adding an auxiliary energy field [8,9], using intermediate layers such as Ni and Zn [10,11,12], cooling by water [13,14] or liquid nitrogen [15]. Among them, ultrasonic vibration (UV) has the advantages of reducing the flow stress of metals during their plastic deformation [16,17], promoting the plastic flow of materials [18], and inhibiting the growth of intermetallic compounds [19,20] in FSW. The UV has been exerted into the FSW process for joining similar and/or dissimilar alloys [21,22].

Scholars have developed different types of ultrasonic-assisted FSW processes, which can be roughly divided into two categories according to the different application modes and locations of UV applied to the tool or the workpiece. As for the way to apply UV to the tool, it can be introduced by bearing transmission at the side [23] or by integrating the UV device with the tool in the axial direction [24,25]. As for the methods applied to the workpiece, they include an introduction at the back of the workpiece [9,26], at one side of the workpiece [20], or in front of the weld nugget zone [27]. UV was introduced into the process of friction stir welding through various UV devices, which has a significant impact on the macro-mechanical properties and microstructure of the weld.

Hu et al. [28] conducted probe penetrating friction stir welding of AA2219-T6 with a thickness of 5 mm with the application of ultrasonic vibration and found that introducing ultrasonic vibration enhanced dynamic recrystallization and precipitation and eliminated the “S” line, thus improving the joint tensile strength and fracture elongation. He et al. [29] studied the coupling effect of axial ultrasonic and tool thread on the 6061-T6 Al/AZ31 Mg FSW lap joint with 3 mm thickness and found the coupling effect of UV and variable pitch thread reduced the intermetallic compounds (IMCs) of the joint and formed an effective mechanical interlocking structure. S. Benfer et al. [21] introduced ultrasonic into the FSW process of AC4800/AZ80 and found that the coherent formation of the brittle intermetallic phase Al_3_Mg_2_ can be suppressed, and an increase of tensile strength of about 25% can be achieved.

In joining dissimilar AA6061/AZ31B alloys, Sachin et al. [30] used UV to assist FSW experiments of the plates with 3 mm thickness and found that the exerted UV reduced the thickness of intermetallic compounds (IMCs) and improved the ultimate tensile strength of the joints. Lv et al. [31] studied the macroscopic morphology and material flow on the cross sections of 3 mm-thick plates with/without UV and found that the UV enhanced the material flow, accelerated the heat dissipation to some extent, and reduced the IMC thickness to weld WNZ. Zhao et al. [27,32] comprehensively studied the effect of ultrasonic on the microstructure and properties of Al-to-Mg joint with 3 mm thickness and found that UV promoted the recrystallization degree and suppressed the growth of IMCs in the weld.

Previous research mainly focused on the joining of dissimilar plates with a thickness of 3 mm or more. Recently, thin Al and Mg sheets have been widely employed in the fields of new energy vehicles, electrical appliances, and 3C products. In FSW of thin sheets, the tool size has to be reduced correspondingly. The smaller shoulder and shorter pin length cause the reduction of heat generation, and the thermal-mechanical process will be changed. Furthermore, the driving effect of the shoulder and pin on the abutting surface of dissimilar materials is weakened, and the volume of WNZ is lowered. In such conditions, the influence of ultrasonic vibration on the macrostructure and mechanical properties of joints will also be changed. Therefore, different process parameters should be used for thinner sheets, and the macrostructure and mechanical properties of joints need to be further explored.

In this study, FSW and UV-enhanced FSW (UVeFSW) experiments were carried out on 6061-T6 and AZ31B-H24 sheets of 2 mm thickness. A suitable process window was found, and the metallography, macrostructure, and mechanical properties of welds in FSW and UVeFSW were analyzed and compared. The mechanism of ultrasonic vibration on the welding process and weld quality of Al/Mg sheets with 2 mm thickness was expounded.

## 2. Experimental Procedure

**Figure 1:**
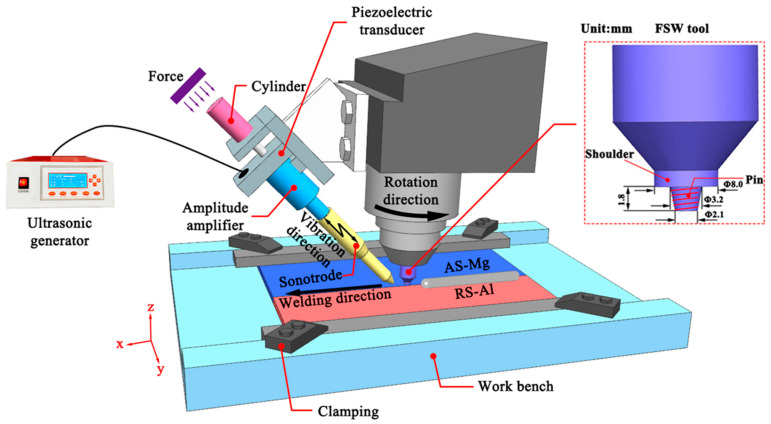
Schematic diagram of ultrasonic vibration enhanced friction stir welding (UVeFSW).

FSW and UVeFSW experiments of 6061-T6 and AZ31B-H24 were conducted. The actual chemical compositions (wt. %) of base metals are as follows: for 6061-T6, Al, (Bal.)-Mg, (0.937)-Cu, (0.245)-Si, (0.66)-Mn, (0.074)-Fe, (0.482)-Zn, (0.08)-Ti, (0.03)-Cr, (0.113); and for AZ31B-H24, Al, (2.97)-Mg, (Bal.)-Cu, (0.005)-Si, (0.03)-Mn, (0.44)-Fe, (0.002)-Zn, (1.26). The ultimate tensile strength (UTS), elongation, and Vickers hardness of 6061-T6 are 283.3 MPa, 11%, and 90 HV, respectively, while those of AZ31B-H24 are 236.7 MPa, 12%, and 55 HV, respectively. The dimensions of the sheets were 200 mm (length) × 70 mm (width) × 2 mm (thickness). The welding direction was the rolling direction of the sheets. As shown in Figure 1, the cylinder pressure provided by the air compressor pushed the sonotrode to contact the surface of the base material smoothly. The included angle between the sonotrode and the horizontal plane was 40°, and the distance between its contact point with the base material and the center of the pin was 20 mm. The output frequency of the ultrasonic generator was 20 kHz, and the maximum working amplitude was about 50 μm. The working amplitude and ultrasonic power can be adjusted by the amplitude output ratio module of the panel, and the adjustment range was 50–100%. In this experiment, the amplitude output ratio was 50%, the working amplitude was about 25 μm, and the ultrasonic power was 260 W ± 10 W.

The tool was made of H13 tool steel and consisted of a concave shoulder and a frustum-shaped pin with right-handed thread. The shoulder diameter was 8 mm, and the pin length was 1.8 mm, in which the top and root diameters were 2.3 and 3.1 mm, respectively. In the welding process, the tool rotated counterclockwise, and the Mg sheet was placed on the advancing side (AS). The pin offset was to the Mg side by 0.4 mm. The plunge depth was 0.15 mm, and the tilt angle of the tool was 2.5°. The tool rotating speed and welding speed were within the ranges of 30–70 mm/min and 800–1200 rpm, respectively.

**Figure 2:**
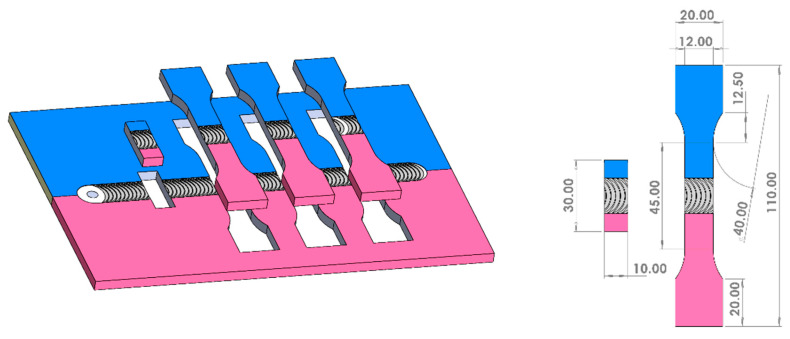
Schematic diagram and detailed size of weld metallographic and tensile specimens.

After welding, the metallographic samples and tensile samples were cut by a wire-cutting machine, as shown in Figure 2. In order to observe the macroscopic metallographic structure of the WNZ, after sanding and polishing, the Mg alloy side was etched by picric acid solution (picric acid 4.2 g + acetic acid 10 mL + distilled water 10 mL + ethanol 70 mL) for 10 s, and the Al alloy side was etched by Keller reagent (hydrofluoric acid 1 mL+ hydrochloric acid 1.5 mL + nitric acid 2.5 mL + distilled water 95 mL) for 5 s. The fracture specimens were first etched by picric acid solution for 10 s and then by sodium hydroxide solution (sodium hydroxide 20 g + distilled water 100 mL) for 60 s. Subsequently, the macroscopic metallography of the WNZ was observed by Zeiss Stemi DV4 stereo-microscope (Carl Zeiss Shanghai Co., Ltd., Shanghai, China), and the material flow interpenetration at the Al/Mg bonding interface was observed by Keyence VHX 500F Optical Microscope (Keyence Corporation, Osaka, Japan). Fracture scanning electron image and energy dispersive spectroscopy (EDS) point scanning analysis were collected and analyzed by JEOL JSM-7800 Scanning Electron Microscope (JEOL(Beijing) Co., Ltd., Beijing, China) and Oxford Instruments XMax80-EDS (Oxford Instruments plc, Abingdon, UK).

The tensile tests were performed by using the Zhongzheng WDW-100AE universal testing machine (Jinan Zhongzheng Testing Machine Manufacturing Co., Ltd., Jinan, China) and the tensile speed of the machine was set to 1 mm/min, and the average value of the three tensile specimens was taken as the ultimate tensile strength of this process parameter. The Vickers hardness distribution of the cross-section of the weld was detected by the Zhongzheng HVS-1000Z-W Vicker Durometer (Jinan Zhongzheng Testing Machine Manufacturing Co., Ltd., Jinan, China). Hardness tests were performed with 0.25 mm for each step in thickness and width direction.

## 3. Results and Discussion

### 3.1. Process Window

According to the previous literature and preliminary experimental research, the shoulder diameter is generally 3–4 times the plate thickness, and the pin length is usually slightly lower than the plate thickness by 0.1–0.2 mm. Therefore, for butt welding of 2 mm thin Al/Mg sheet, a tool with smaller shoulder size and shorter pin length was adopted compared with the widely studied 3 mm and above medium plate, so the thermal-mechanical coupling process in the welding process is bound to change. Therefore, it is necessary to carry out experiments to explore the FSW and UVeFSW process windows for Al/Mg sheets of 2 mm thickness. Within the range 800–1200 rpm, the rotation speed was taken every 100 rpm, while within the range 30–70 mm/min, the welding speed was taken every 10 mm/min. Finally, according to whether the weld was smooth and free-defects, the welding process windows of FSW and UVeFSW were determined.

**Figure 3:**
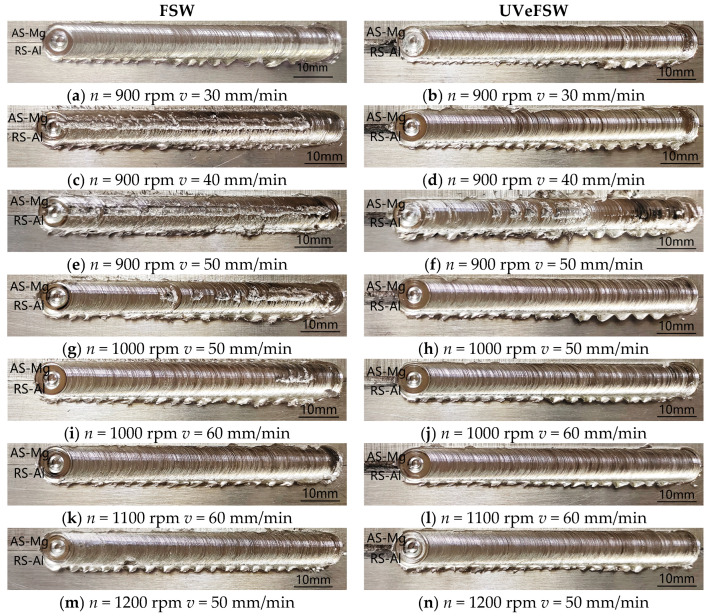
Surface morphology of FSW/UVeFSW welds.

When the tool rotation speed was 800 rpm, no matter what welding speed was adopted, a well-formed joint could not be obtained. Figure 3 shows the weld surface under some typical parameters. When the process parameter set was 900 rpm and 30 mm/min, the joints made by FSW and UVeFSW were well formed. When the welding speed was increased to 40 mm/min, obvious groove defects appeared on the FSW surface due to lack of heat input. At the same parameter set, UVeFSW can obtain a well-formed weld, which shows that ultrasonic vibration can enhance the plastic flow of the material around the pin by reducing the material flow stress so that the material can fill the instantaneous cavity behind the moving path of the tool. When the welding speed was 50 mm/min, the welding heat input was further reduced, and long and deep grooves were detected on the weld surface in FSW, but there were only some hole defects in UVeFSW. When the tool rotating speed was 1000 rpm, due to the increase of heat input, there were no obvious groove defects in FSW/UVeFSW processes, which showed that the heat input was reasonable within this parameter range. At the welding speed of 50–70 mm/min, the FSW weld had material loss caused by plastic material “sticking” to the pin because the plastic material was taken away and deposited by the stirring pin before it could fully fill the instantaneous cavity of the stirring pin. However, the surface of the UVeFSW weld was well formed, and there was no “adhesion” phenomenon. This was because ultrasonic vibration enhanced the flow ability of plastic materials, so the “adhesion” phenomenon struggled to appear. When the rotation speed was 1100 rpm or 1200 rpm, the weld with good surface formation can be obtained in two processes within the welding speed range of the experiment.

**Figure 4:**
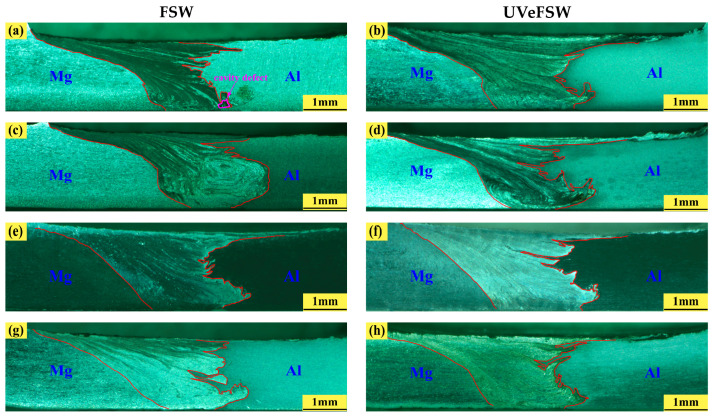
Macrographs of welds at transverse cross-section under typical parameters. (**a**) *n* = 900 rpm *v* = 30 mm/min. (**b**) *n* = 900 rpm *v* = 30 mm/min. (**c**) *n* = 1000 rpm *v* = 40 mm/min. (**d**) *n* = 1000 rpm *v* = 40 mm/min. (**e**) *n* = 1200 rpm *v* = 50 mm/min. (**f**) *n* = 1200 rpm *v* = 50 mm/min. (**g**) *n* = 1100 rpm *v* = 60 mm/min. (**h**) *n* = 1100 rpm *v* = 60 mm/min.

Figure 4 shows the macrographs of welds at transverse cross-section under typical parameters. When the process parameter set was 900 rpm–30 mm/min, tiny hole defects appeared at the bottom of the FSW weld, as shown in Figure 4a. The low rotation speed led to less heat generated between the tool and the base metal, which led to the low temperature at the bottom of the weld and poor plastic flow ability of the material, and it was difficult to deposit on the path of pin moving, thus forming hole defects. Under the same parameters, there was no hole defect at the bottom of the UVeFSW weld, which showed that the application of ultrasonic vibration can improve the material flow so that the material at the bottom of the weld can flow more fully, even at relatively low temperatures.

Under typical parameters that can obtain well-formed and free-defect joints, such as 1000 rpm–40 mm/min, 1200 rpm–50 mm/min, and 1100 rpm–60 mm/min, it can be found that more “interspersed strips” and “hook-shaped structure” in the cross-section of the weld with ultrasonic assistance as shown in Figure 4d,f,h. That was because the acoustic softening effect of ultrasonic reduced the flow stress of the base metal and promoted the material flow interpenetration [33]. Those zigzag structures made the interface of Al and Mg more complicated and tortuous. Thereby, the mechanical interlocking degree of Al and Mg materials was improved.

**Figure 5:**
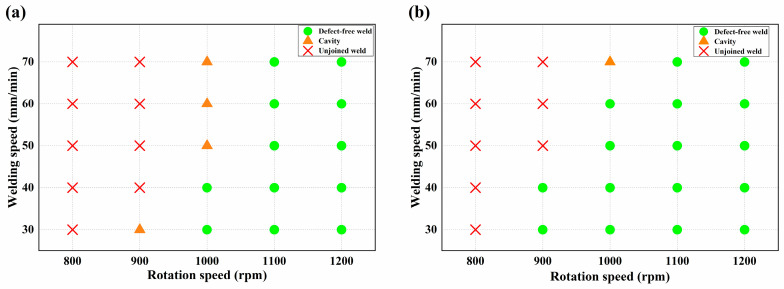
Process parameter windows of 2 mm thickness 6061-T6/AZ31B-H24 dissimilar (**a**) FSW/(**b**) UVeFSW.

To sum up, the application of ultrasonic vibration improved the material flow, reduced the welding defects inside the weld, and broadened the welding process window, as shown in Figure 5. This would be of great significance to achieve sound dissimilar joints of Al/Mg alloys in industrial production.

### 3.2. Mechanical Interlocking

The interpenetration of materials on the bonding interface reflects the degree of mechanical interlocking of dissimilar joints, which is one of the important factors affecting the mechanical properties of joints. Therefore, with the process parameter set 1200 rpm–50 mm/min in FSW/UVeFSW, the material flow and mechanical interlocking in the upper, middle-upper, middle-lower, and lower regions on the transverse cross-section of the welds were further characterized.

**Figure 6:**
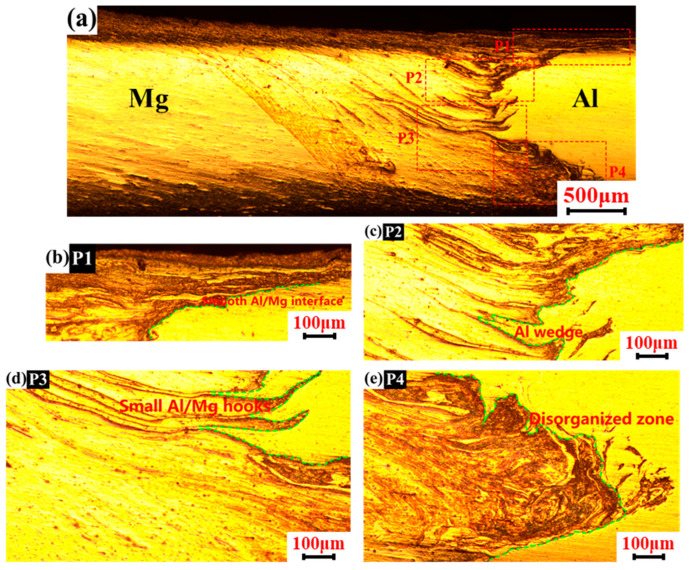
Macrographic images of FSW weld on transverse cross-section (*n* = 1200 rpm, *v* = 50 mm/min). (**a**) location overview; (**b**) upper region; (**c**) middle-upper region; (**d**) middle-lower region; (**e**) lower region.

Figure 6 shows the metallographic images of the FSW weld at transverse cross-section. And the interrupted green lines depict the Al and Mg boundary. In the upper P1 region, the bonding interface between Al and Mg was smooth, and there were many Al alloy strips surrounded by Mg alloy at the top, which was because the material at the top was driven by the shoulder, and the material flow was more intense. At the top of the P2 region, the interface was still relatively flat. But at the bottom of the P2 region, there were more curved Al alloy strips inserted into the Mg alloy matrix to form a wedge-shaped insertion structure. In the P3 region, several Al/Mg strips were obviously interspersed with each other. Although the Al alloy strips were deeply interspersed, the strips were very small. In the P4 region, the intertwining phenomenon of Al/Mg materials was very distinct, and the material flow was very chaotic. At the top of the P4 region, a small “hook” structure was formed. In the middle of the P4 region, many independent and discontinuous Mg alloy strips were formed. At the bottom of the P4 region, the Al/Mg interface was very flat, which was due to the small diameter of the pin tip and the weak driving ability of the material here.

**Figure 7:**
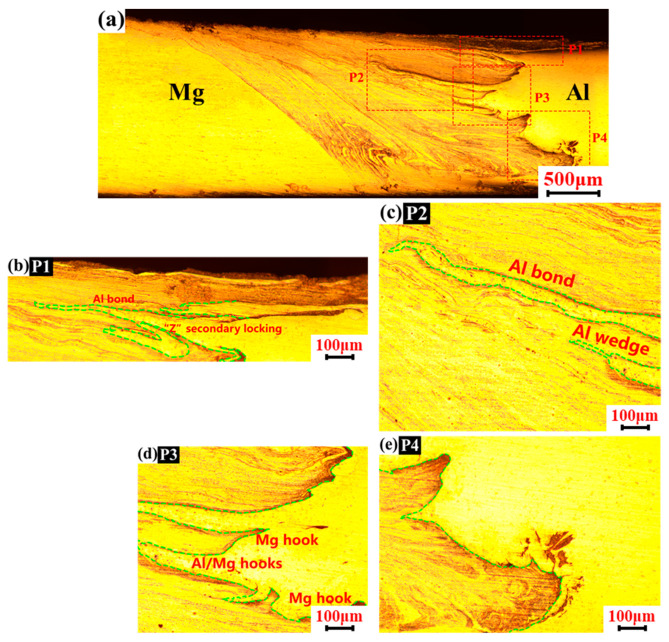
Macrographic images of UVeFSW weld on transverse cross-section (*n* = 1200 rpm, *v* = 50 mm/min). (**a**) location overview; (**b**) upper region; (**c**) middle-upper region; (**d**) middle-lower region; (**e**) lower region.

Figure 7 shows the metallographic images of the UVeFSW weld at the transverse cross-section. In the middle of the P1 region, Al/Mg strips with deep interpenetration formed, and a mechanical interlocking structure similar to “Z” was formed. Compared with the FSW-P1 region in Figure 6, when ultrasonic vibration was applied, the material flow at the top of the WNZ was smoother. Also, the formed Al/Mg strips were deeper, and the mechanical interlocking degree was obviously enhanced with application of ultrasonic.

In the P2 region, Al alloy strips were greatly inserted into the Mg matrix, and a small “hook” interlocking structure was formed at the end of the strip. Moreover, the interspersed Al alloy strips were thicker, which had a strong effect on preventing crack propagation. In the P3 region, compared with the FSW-P3 region in Figure 6, UVeFSW weld had formed Al/Mg strips with greater penetration and greater thickness. This kind of thick-strip penetration was different from thin-strip penetration. The former can effectively prevent extending the crack propagation path, while the latter is difficult to hinder the crack propagation. At the same time, a small “hook” interlocking structure and Al/Mg strips inserting structure were also formed at the bottom of the P3 region, which showed that applying ultrasonic vibration can promote the material flow and promote the Al/Mg strips to form more and larger inserting structures and interlocking structures, which was beneficial to improve the ultimate tensile strength of the joint. In the middle and upper part of the P4 region, a smaller “hook” structure was formed. Discontinuous “free” Mg alloy strips were also formed at the bottom of the P4 region, and the Al/Mg bonding interface in the P4 region was relatively smooth on the whole.

To sum up, the application of ultrasonic vibration made the Al/Mg strips form more “wedge-shaped” interpenetrating structures and “hook-shaped” interlocking structures with larger size, which made the Al/Mg bonding interface more tortuous and enhanced the mechanical interlocking degree.

### 3.3. Width of WNZ

**Figure 8:**
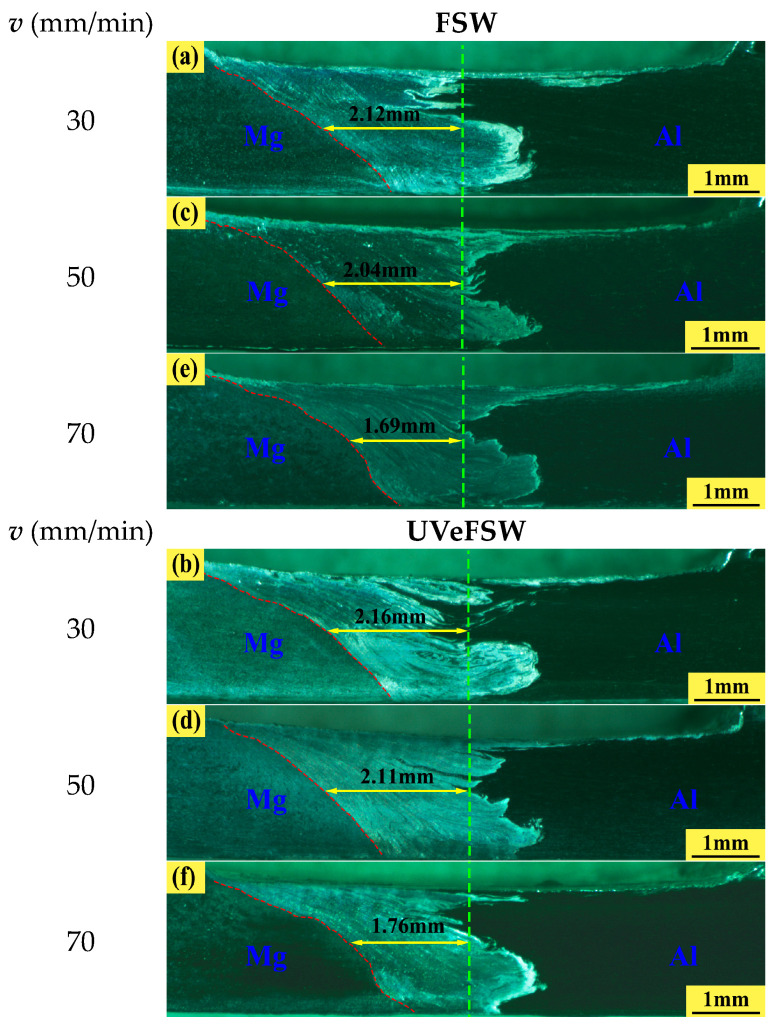
WNZ width in FSW/UVeFSW welds at middle depth (*n* = 1200 rpm) under different welding speeds. (**a**,**c**,**e**) FSW; (**b**,**d**,**f**) UVeFSW. (**a**,**b**) 30 mm/min; (**c**,**d**) 50 mm/min; (**e**,**f**) 70mm/min.

Figure 8 shows the WNZ width in FSW/UVeFSW welds at mid-depth on the transverse cross-section. The green dotted line is the axis of the pin, and the horizontal position of the yellow solid line is 1 mm away from the bottom of the weld, that is, half the thickness of the plate. On the advancing side (Mg), the tangential direction of the rotation speed of the tool is consistent with the direction of welding speed so that the Mg alloy in the WNZ on the advancing side was more fully sheared and stirred by the tool and its grain size and orientation were quite different from those in the thermo-mechanical affected zone (TMAZ), so that the boundary between the WNZ and the TMAZ was clear. On the retreating side (Al), the tangential direction of the rotation speed of the tool was opposite to the direction of welding speed, which made the material in the WNZ on the retreating side mainly squeezed by the tool, and the difference in grain size and orientation between WNZ and TMAZ was not obvious compared to that at the advancing side, which made its boundary relatively inconspicuous. Therefore, for convenience, the distance from the axis of the pin to the boundary line of TMAZ-WNZ on the advancing side was defined as the half-width of the WNZ. By comparison, it can be seen that under the same process parameters, the half-width of the WNZ in UVeFSW was a little bit larger than that in FSW because ultrasonic vibration can reduce the deformation resistance of the material and enhance the plastic flow ability of the materials. The ultrasonic vibration can enlarge the width of WNZ under the condition of medium and low heat input, and the half-width of WNZ decreases slowly with the increase of welding speed. Because the ultrasonic power used was low, it only played an auxiliary role, so it only enhanced the material flow to a certain extent. According to the obtained data, the average half-width of WNZ was increased by about 5% in UVeFSW.

### 3.4. Hardness Distribution

**Figure 9:**
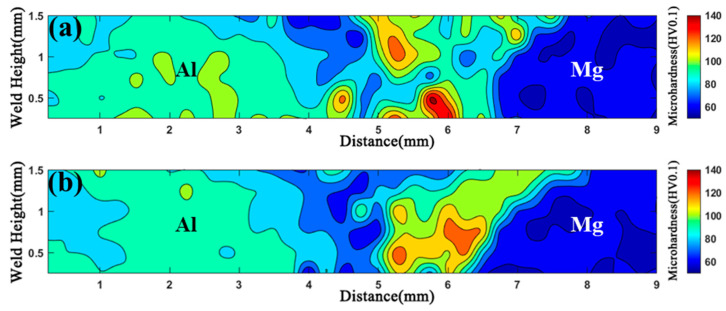
Hardness maps of the joint cross-sections (1200 rpm–50 mm/min). (**a**) FSW, (**b**) UVeFSW.

Figure 9 shows the hardness map on the weld cross-section of the FSW and UVeFSW joints at 1200 rpm–50 mm/min. The hardness was tested every 0.25 mm in both the thickness and width direction of the weld. The hardness type was Vickers hardness. It can be seen that the banded zone on the advancing side of FSW and UVeFSW joints had high hardness. Compared with the FSW joint, the hardness distribution of the UVeFSW joint was more uniform, the highest hardness and average hardness were relatively reduced, and the low hardness zone in WNZ was slightly enlarged.

6061-T6 alloys are commonly used Al–Mg–Si alloys. Metastable phase precipitation occurs during heat and/or thermomechanical treatments in the Al–Mg–Si alloys and influences their degree of performance. The hardening phases are the metastable precursors of the stable Mg_2_Si phase. The precipitation sequence in Al–Mg–Si alloys that is generally accepted is as follows [34,35]: SSSS (super-saturated solid solution) → solute clusters → GP-zones (spherical) → β″-Mg_5_Si_6_ → β′-Mg_1.8_Si + U1-MgAlSi + U2-MgAlSi + B′-Mg3Al9Si7 → β-Mg2Si + Si (stable). Distinction between β′, U1, U2, B′ phases is not evident; thus, we only use β′ to represent them. With the process of over-aging, the metastable phase will change to a stable Mg_2_Si phase, which will further reduce the strength and hardness and improve the plasticity and toughness of the alloy.

In addition, the hardness of Al alloy located in WNZ was lower than that of Al alloy outside WNZ, which might be due to the higher temperature in WNZ, which led to the dissolution of β″ phase in the base metal in Al alloy matrix, or the transformation of β″ phase in the base metal after solution treatment and artificial aging into β′ phase or β phase, and then the hardness decreased due to over-aging. The middle and lower parts of WNZ had higher local hardness than the middle and upper parts, which may be related to the temperature in different areas. The middle and upper parts near the shoulder generated more heat, which may lead to the dissolution of the second phase of Al alloy and the coarsening of grains compared with the bottom.

### 3.5. Ultimate Tensile Strength

**Figure 10:**
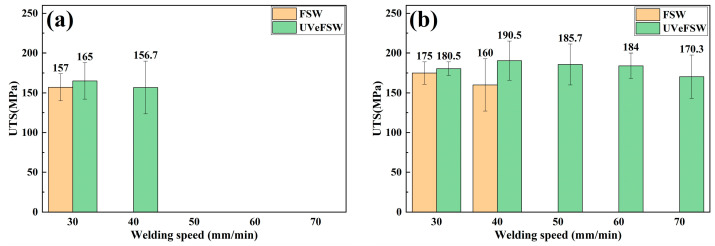

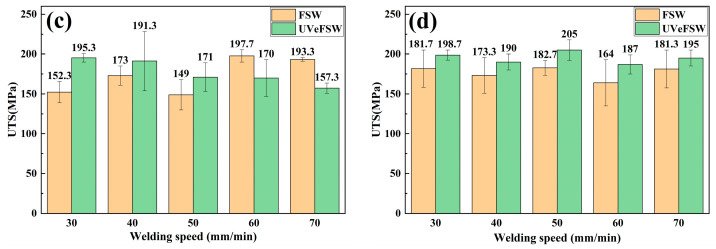
Ultimate tensile strength of FSW/UVeFSW joints. (**a**) *n* =900 rpm. (**b**) *n* =1000 rpm. (**c**) *n* =1100 rpm. (**d**) *n* =1200 rpm.

Figure 10 shows the ultimate tensile strength of FSW and UVeFSW joints at different process parameters. Compared with FSW joints, the ultimate tensile strength of UVeFSW joints had been increased. Among them, the highest ultimate tensile strength of the FSW joint was 197.7 MPa at 1100 rpm–60 mm/min. The highest ultimate tensile strength of the UVeFSW joint was 205 MPa at 1200 rpm–50 mm/min. The maximum tensile strength increased by about 3.7% when ultrasonic vibration was applied. Considering all the parameters, the ultimate tensile strength of tensile specimens under 13 groups of parameters was averaged again. The average ultimate tensile strength of the FSW and UVeFSW joint was 172.3 MPa and 184.4 MPa, respectively. When ultrasonic vibration was applied, the average ultimate tensile strength of tensile specimens under 13 groups of parameters was increased by 7.02%.

### 3.6. Fracture Analysis

**Figure 11:**
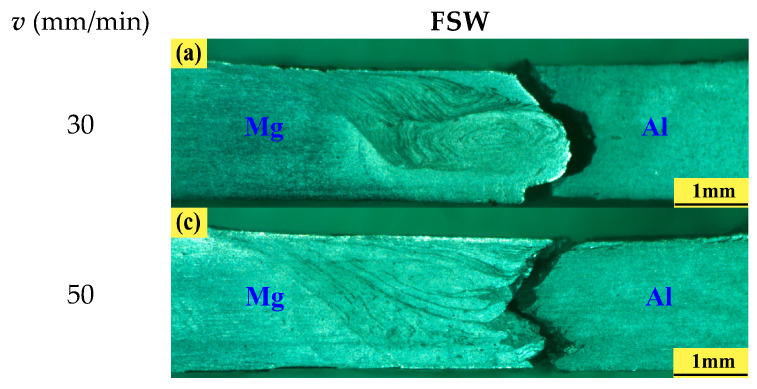

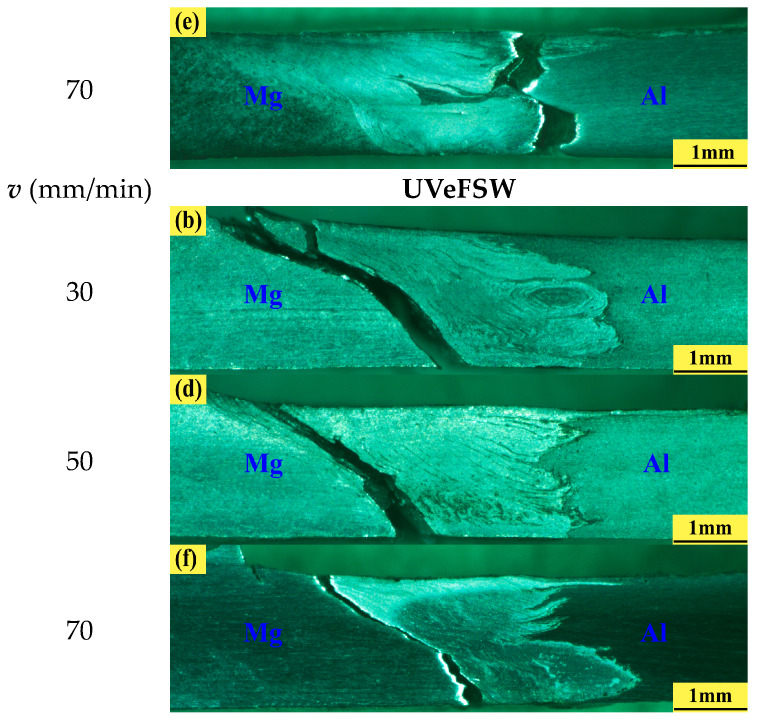
Fracture positions of FSW/UVeFSW joints (*n* = 1200 rpm) under different welding speeds. (**a**,**c**,**e**) FSW; (**b**,**d**,**f**) UVeFSW. (**a**,**b**) 30 mm/min; (**c**,**d**) 50 mm/min; (**e**,**f**) 70mm/min.

Figure 11 shows the fracture positions of FSW and UVeFSW joints at 1200 rpm. It can be seen that the FSW joints were generally fractured at the Al/Mg bonding interface, while the UVeFSW joints were all fractured at the TMAZ-WNZ boundary on the advancing side. Ultrasonic vibration changed the fracture position of welded joints, which was mainly due to the more tortuous Al/Mg bonding interface in UVeFSW and the formation of more and larger “hook” interlocking structures and “wedge” interspersed structures. In addition, in some cases, FSW joints did not fracture strictly along the Al/Mg bonding interface; that was, they did not fracture completely at the interspersed Al/Mg strips but were divided into two situations: (1) When the cracks started from the bottom of the joint and spread to the top along the smooth Al/Mg bonding interface at the bottom, once they encountered the interspersed Al/Mg strips, the cracks did not turn. Instead, the cracks passed directly through Al/Mg strips under increasing tensile force, continuing to propagate along the interface until complete fracture occurred, as shown Figure 11c. This showed that the tiny Al/Mg interpenetrating structure cannot effectively change the crack propagation path. (2) When the crack also started from the bottom of the joint spread to the top along the smooth Al/Mg bonding interface at the bottom of the joint, once the Al/Mg strips with deep penetration were encountered, the crack will not propagate along the Al/Mg bonding interface, but will suddenly break at the end of the strip where the Mg strip was inserted into the Al alloy, that was, it will break on the Al alloy side, and the crack will continuously expand upward and then break, as shown in Figure 11a,e. This showed that the interpenetration of larger Al/Mg strips could effectively hinder the crack propagation path and proved the importance of mechanical interlocking structure to the fracture position of welded joints.

The fracture morphology of the joints under different welding process parameters was observed by scanning electron microscope (SEM). The fracture morphology can reflect the fracture mode and other information of the joints.

**Figure 12:**
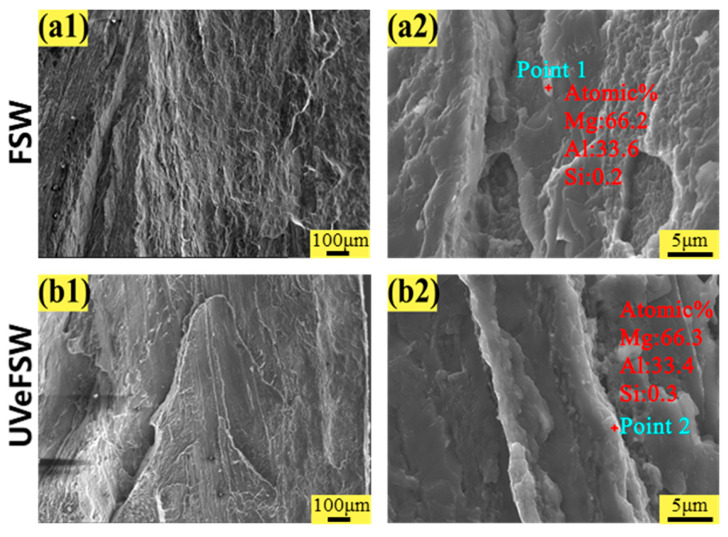
Fracture SEM images of tensile specimens (*n* = 1200 rpm, *v* = 30 mm/min). (**a1**,**a2**) FSW, (**b1**,**b2**) UVeFSW.

Figure 12 shows the fracture morphology of FSW and UVeFSW welds at 1200 rpm–30 mm/min. For the fracture of the FSW joint, the whole fracture surface was very flat and smooth, and an obvious river pattern can be seen, which was a distinct symbol of brittle fracture. Under the same process parameters, the fracture morphology of the UVeFSW joint also showed obvious brittle fracture behaviors. Subsequently, the fracture surfaces were tested by SEM-EDS point analysis, and the results are shown in Table 1 and Figure 12(a2,b2). From the point analysis data and fracture scanning electron image, it can be known that there were Al-Mg intermetallic compounds on the fracture surface at 1200 rpm–30 mm/min in the FSW and UVeFSW process. Because the Al–Mg intermetallic compounds are brittle IMCs with high hardness, it is easy to cause stress concentration in the joint and lead to cracks and extend to fracture.

**Figure 13:**
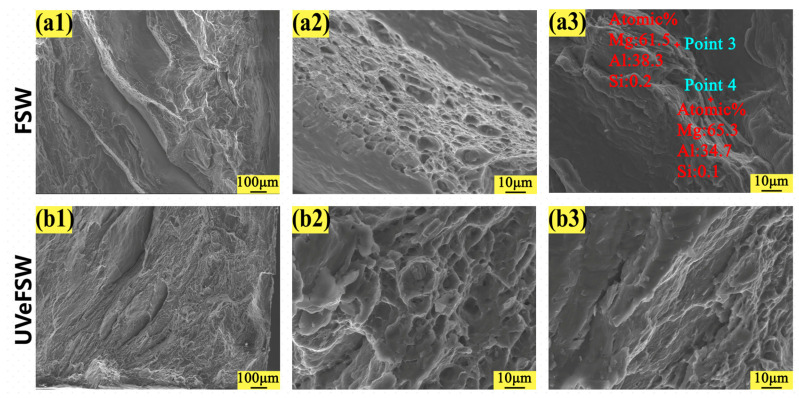
Fracture SEM images of tensile specimens (*n* = 1200 rpm, *v* = 50 mm/min). (**a1**–**a3**) FSW, (**b1**–**b3**) UVeFSW.

**Figure 14:**
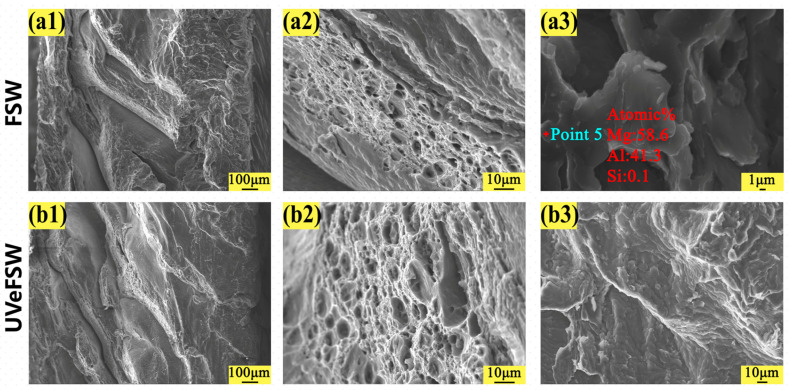
Fracture SEM images of tensile specimens (*n* = 1200 rpm, *v* = 70 mm/min). (**a1**–**a3**) FSW, (**b1**–**b3**) UVeFSW.

The fracture morphology of the joints at 1200 rpm–50 mm/min and 1200 rpm–70 mm/min were shown in Figure 13 and Figure 14, respectively. On the whole, there were obvious river patterns and a large number of dimple areas in the fracture morphology of FSW/UVeFSW joints, which were judged as ductile-brittle mixed fractures. In the same visual field, the fracture dimple size of UVeFSW joints was larger and deeper, which indicated there were larger ductile fracture areas of the UVeFSW joints. It also meant that the plasticity of the UVeFSW joints was better. As the data in Table 1 and Figure 13(a3) and Figure 14(a3) show, point analysis data still show that there are Al/Mg IMCs on the fracture interface, which once again proves that the IMCs in the Al/Mg dissimilar joint are the main reason for the fracture in the FSW process. In addition, IMCs were not found at the surface of fracture at 1200 rpm–50 mm/min and 1200 rpm–70 mm/min in UVeFSW. Compared with the fracture at 1200 rpm–30 mm/min in UVeFSW, the heat input is relatively reduced at the welding speed of 50/70 mm/min with the same rotation speed, and the growth and distribution of IMCs have also changed. The results show that it was not generated at the TMAZ-WNZ boundary on the advancing side at 1200 rpm–50 mm/min and 1200 rpm–70 mm/min in UVeFSW, which further improves the strength of the joint.

## 4. Conclusions

(1) The FSW/UVeFSW process parameters windows were determined for joining 6061-T6/AZ31B-H24 sheets of thickness 2 mm at the rotation speed of 800–1200 rpm and the welding speed of 30–70 mm/min. Under the same welding conditions, a larger process parameters window was obtained with the application of ultrasonic vibration;
(2) Ultrasonic vibration improved the formation quality at the weld surface and interior by reducing the deformation resistance of the materials and promoting the plastic material flow. Compared with the FSW joint, the hardness distribution of the UVeFSW joint was more uniform, the highest hardness and average hardness were relatively reduced, and the low hardness zone in the WNZ was slightly enlarged. Applying ultrasonic vibration can effectively reduce the weld defects, such as grooves and holes on the weld surface and interior, and increase the width of the WNZ;
(3) The application of ultrasonic vibration promoted the formation of more hook-shaped interlocking structures and wedge-shaped interpenetrating structures of Al/Mg strips, which made the Al/Mg bonding interface more tortuous and enhanced the mechanical interlocking degree. The maximum ultimate tensile strength of welded joints was increased from 197.7 MPa in FSW to 205 MPa in UVeFSW, and the increased range was about 3.7%. The average ultimate tensile strength of 13 groups of welded joints with different welding process parameters increased from 172.3 MPa in FSW to 184.4 MPa in UVeFSW, with an increase rate of about 7.02%;
(4) The fracture positions of the joints were changed from the Al/Mg bonding interface to the vicinity of the boundary between the WNZ of the advancing side and the TMAZ with ultrasonic vibration. EDS data analysis showed that no IMCs were found in the fracture of Al-Mg joints at 1200 rpm–50/70 mm/min in the UVeFSW process, which indicated that the application of UV had an impact on the formation and distribution of intermetallic compounds. Applying ultrasonic vibration also made the number and size of dimples on the fracture surface larger, which improved the plasticity of the joint.

## Figures and Tables

**Table 1:** SEM-EDS point analysis data of the fractures.

Point Number	Parameter	Mg (%)	Al (%)	Si (%)
1	FSW-1200-30	66.2	33.6	0.2
2	UVeFSW-1200-30	66.3	33.4	0.3
3	FSW-1200-50	61.5	38.3	0.2
4	FSW-1200-50	65.3	34.7	0.1
5	FSW-1200-70	58.6	41.3	0.1

## Data Availability

The original contributions presented in the study are included in the article, further inquiries can be directed to the corresponding author.
